# Therapeutic Notes

**Published:** 1898-07

**Authors:** W. J. Martin

**Affiliations:** Kankakee, Ill.


### THERAPEUTIC NOTES.

CONDUCTED BY W. J. MARTIN, V.S.,
KANKAKEE, ILL.

THE COMPARATIVE VALUE OF ANTISEPTICS. A. Gawalowski has brought together in tabulated form a statement of the comparative value of a number of antiseptics, ranking them according to their efficiency as disinfectants, antiseptics, and deodorizers. In this table corrosive sublimate is taken as a standard for comparison, with a value of 100.

| Substance. | Disinfectant. | Antiseptic. | Deodorizer. |
|---|---|---|---|
| Corrosive sublimate . . . . | 100 | 100 | ... |
| Carbolic acid (100 per cent.) . . | 50 | 50 | 40 |
| Sulphurous acid (gas) . . . | 50 | 50 | 50 |
| Thymol (solid) . . . . . | 50 | 50 | 40 |
| Creosote (100 per cent.) . . | 50 | 50 | 40 |
| Antimony (Bayer) . . . . | 50 | 50 | 40 |
| Iron vitriol (crystal) . . . | 40 | 50 | 30 |
| Ferrous zinc sulphate (crystal) . | 40 | 50 | 40 |
| Iron oxide . . . . . . | 40 | 10 | 15 |
| Ferrous zinc magnesium sulphate (crystal) . . . . . . | 40 | 30 | 30 |
| Copper zinc sulphate (crystal) . | 35 | 40 | 40 |
| Ferrous zinc cupric sulph. (cryst.) | 35 | 40 | 40 |
| Zinc phenolate (dry) . . . | 30 | 40 | 45 |
| Sulphites (10–40 per cent.) . | 15–25 | 15–25 | 15–25 |
| Permanganates (solid) . . . | 25 | 10 | 50 |
| Salicylates (solid) . . . . | 25 | 25 | 40 |
| Creolin . . . . . . . | 25 | 25 | 40 |
| Lysol . . . . . . . | 25 | 25 | ... |
| Chlorine (gas) . . . . . | 15 | ... | 50 |
| Ferrous zinc calcium sulph. (cryst.) | 15 | 30 | 40 |
| Ferrous salts (40–50 per cent. sol.) | 10 | 10 | 5 |
| Zinc salts (crystal) . . . | 10 | ... | 40 |
| Calcium chloride . . . . | 5 | ... | 15 |

—*Pharm. Post and Phar. Review.*

INTERNAL ADMINISTRATION OF CREOSOTE. Creosote is best administered to small animals in a capsule containing equal parts of olive oil and balsam-of-tolu. This mixture does not irritate the digestive tracts, and exercises also a direct therapeutical effect upon the bronchial mucous membranes. For administration to horses and cattle, creosote should be mixed with milk, with which it forms a perfect emulsion.

To Destroy Worms in Powdered Vegetable Drugs. Place the infected drug in an air-tight bottle and place therein a small sponge or piece of felt saturated with pure chloroform. The chloroform quickly destroys all insect-life, and can be removed from the drug by simply leaving the stopper of the bottle out for a short time after removing the sponge or felt.

Healing Salves.

| | | |
|---|---|---|
| No. 1. | ℞.—Lanolin | 125 parts. |
| | Vaselin | 350 " |
| | Glycerin | 350 " |
| | Zinc oxide | 125 " |
| | Carbolic acid | 10 " |

Mix the lanolin and vaselin, add the glycerin and work together, then add the zinc oxide and carbolic acid, and work until thoroughly incorporated.

| | | |
|---|---|---|
| No. 2. | ℞.—Lanolin | 4 parts. |
| | Vaselin | 350 " |
| | Boro glyceride, 50 per cent. | 4 " |
| | Glycerin | 8 " |

Mix as above.

| | | |
|---|---|---|
| No. 3. | ℞.—Lanolin | 125 parts. |
| | Vaselin | 350 " |
| | Glycerin | 350 " |
| | Zinc oxide | 125 " |
| | Tannin | 8 " |

Mix as above.

An Application for Insect-bites and Stings.

| | | |
|---|---|---|
| No. 1. | ℞.—Olive oil | 4 ounces. |
| | Aqua ammonia | 4 " |
| | Oil turpentine | 2 drachms. |
| | Tinct. opium | 2 " |

Mix.

| | | |
|---|---|---|
| No. 2. | ℞.—Carbolic acid | 4 drachms. |
| | Glycerin | 2 ounces. |
| | Water | 4 drachms. |

Mix by aid of a gentle heat.

—*Bull. Pharmacy.*

What is Medicine? We have been frequently at a loss to decide what constitutes a drug or medicine, but the Pharmaceutical Congress at Brussels has settled the question for us, and declares that "By medicine we understand any substances to which is attributed the property of being able to restore men and animals to a state of health." This is sufficiently comprehensive and very carefully worded. Now, what are we to call substances to which is attributed the faculty of being able to endanger the health of men and animals? And as we are told that this is an age of preventive medicine, what are we to call those agents which are employed for the purpose of preventing injury to the health of men and animals?—*Bull. Pharmacy.*

Benzoated Lard can be economically prepared by melting in a water-bath one pound of fresh lard, and incorporating with it five drachms of finely-powdered gum-benzoin. A more elegant preparation may be had by substituting benzoic acid for the benzoin.

Olive Oil is an excellent dressing for bruises, swellings, and collar-excoriations so often met with in horses.

To Remove Warts. A concentrated solution of bichromate of potassium applied once a day will effectually remove these troublesome growths so often met with in veterinary practice.

An Explosive Compound. A combination of iodine and oil of turpentine is incompatible, and is liable to form a dangerous explosive mixture.

---

The proceedings of the National Livestock Association of the United States is added to in value by contributions from Veterinarians Gresswell, of Colorado; Clement, of Baltimore, Md.; Peters, of Nebraska; Norgard, of Washington; and Knowles, of Helena, and their contributions are further embellished with photogravure reproductions of the authors.

The total expenditure on rinderpest in Natal, South Africa, up to March 31, 1898, was £187,023 12s. 11d. It is estimated that of this a sum of £25,000 will be repaid into the treasury, leaving the actual expenditure at approximately £162,000.

Prof. Henry, of Wisconsin, says the weight of evidence is against feeding cooked food to hogs; they thrive best on uncooked food.

The oyster is one of the strongest of creatures, and the force required to open it is more than 1300 times its own weight.

Neither camels nor elephants can jump.

